# JAK3 Y841 Autophosphorylation Is Critical for STAT5B Activation, Kinase Domain Stability and Dimer Formation

**DOI:** 10.3390/ijms241511928

**Published:** 2023-07-25

**Authors:** Georgialina Rodriguez, George Steven Martinez, Omar Daniel Negrete, Shengjie Sun, Wenhan Guo, Yixin Xie, Lin Li, Chuan Xiao, Jeremy Aaron Ross, Robert Arthur Kirken

**Affiliations:** 1 Department of Biological Sciences, The University of Texas at El Paso, 500 W University Ave., El Paso, TX 79968, USA; 2 Border Biomedical Research Center, The University of Texas at El Paso, 500 W University Ave., El Paso, TX 79968, USA; 3 Department of Physics, The University of Texas at El Paso, 500 W University Ave., El Paso, TX 79968, USA; 4 Computational Science Program, The University of Texas at El Paso, 500 W University Ave., El Paso, TX 79968, USA; 5 Department of Biochemistry, The University of Texas at El Paso, 500 W University Ave., El Paso, TX 79968, USA

**Keywords:** Janus Kinase (JAK), Signal transducer and activators of transcription 5 (STAT5), phosphotyrosine signaling, protein structure, molecular dynamic simulations, folding energies, biomolecule interactions

## Abstract

Janus tyrosine kinase 3 (JAK3) is primarily expressed in immune cells and is needed for signaling by the common gamma chain (γc) family of cytokines. Abnormal JAK3 signal transduction can manifest as hematological disorders, e.g., leukemia, severe combined immunodeficiency (SCID) and autoimmune disease states. While regulatory JAK3 phosphosites have been well studied, here a functional proteomics approach coupling a JAK3 autokinase assay to mass spectrometry revealed ten previously unreported autophosphorylation sites (Y105, Y190, Y238, Y399, Y633, Y637, Y738, Y762, Y824, and Y841). Of interest, Y841 was determined to be evolutionarily conserved across multiple species and JAK family members, suggesting a broader role for this residue. Phospho-substitution mutants confirmed that Y841 is also required for STAT5 tyrosine phosphorylation. The homologous JAK1 residue Y894 elicited a similar response to mutagenesis, indicating the shared importance for this site in JAK family members. Phospho-specific Y841-JAK3 antibodies recognized activated kinase from various T-cell lines and transforming JAK3 mutants. Computational biophysics analysis linked Y841 phosphorylation to enhanced JAK3 JH1 domain stability across pH environments, as well as to facilitated complementary electrostatic JH1 dimer formation. Interestingly, Y841 is not limited to tyrosine kinases, suggesting it represents a conserved ubiquitous enzymatic function that may hold therapeutic potential across multiple kinase families.

## 1. Introduction

The common gamma chain (γc) cytokine Interleukin-2 (IL-2) is a functionally important growth factor necessary for T-cell activation and expansion [1,2]. Unlike tyrosine kinase receptors, γc receptors lack intrinsic enzymatic activity and thus associate with Janus tyrosine kinase 1 (JAK1) and JAK3 to initiate intracellular signaling cascades [1]. Upon cytokine binding of the IL-2R complex, JAK3 associated with the γc forms heterodimers with JAK1 to phosphorylate residues localized to the IL-2Rβ subunit [3]. Subsequently, IL-2Rβ acts as a docking site for SH2-containing proteins, including Signal Transducer and activator of transcription 1 (STAT1), STAT3, and STAT5 [3,4,5]. STATs become activated via tyrosine and serine phosphorylation, and disengage from the receptor to form homo- or heterodimers prior to translocation to the nucleus in order to promote the expression of pro-survival and proliferative genes [6]. For an in-depth review of canonical cytokine/JAK/STAT signaling cascade, we refer readers to Morris, et al. 

JAK family members, namely JAK1, JAK2, JAK3, and tyrosine kinase-2 (TYK-2) possess four functional domains: an N-terminal FERM (band 4.1, Ezrin, Radixin, Moesin) domain, an atypical SH2, a pseudokinase, and a catalytically active kinase domain across seven shared Janus homology (JH) domains [7]. The JAK FERM and SH2-like domains span JH4-7 regions and collaborate to bind cytoplasmic motifs within the cytokine receptor [8,9,10]. The pseudokinase domain, confined to the JH2 region, binds ATP and a cation required for phosphoryl transfer, but lacks catalytic activity [11]. The pseudokinase domain is important for the regulation of kinase activity [12,13,14,15,16]. The C-terminal JH1 domain houses the catalytically active motif responsible for the transfer of γ-phosphate from ATP to substrate tyrosine residues [6,17].

Several phospho-tyrosine sites have been identified within JAK3, which can negatively or positively regulate catalytic function. The phosphorylation of tyrosine residue 785 (Y785), located within the linker region between the JH1 and JH2 domain, in response to IL-2 and IL-15 [18] has been observed to control 50% of JAK3 total tyrosine phosphorylation [19]. However, despite reduced phosphorylation, Y785F does not affect its catalytic activity. While SH2B-β and SH2B-3 have been identified as regulators of JAK2 and JAK1 signaling, neither have been reported to impact JAK3 function more than serving as an adaptor protein [20]. Two additional JH1 sites, Y980 and Y981, located within the kinase activation loop, were identified using in vitro kinase assays [21]. Classically, the phosphorylation of residues within the kinase activation loop is associated with promoting full catalytic activity [22,23]. Phospho-substitution mutations established Y980 as a positive regulator that is necessary for STAT5 activation, since an Y980F mutant resulted in decreased IL-2-induced STAT5 DNA binding [21]. Conversely, the Y981F mutant increased STAT5’s DNA-binding activity [21] and conceivably played a role in negative kinase activity. The phosphorylation of the JAK3 JH1 domain residues Y904 and Y939 was determined to function as a positive regulator necessary for optimal autophosphorylation, STAT5 tyrosine phosphorylation and transcriptional activity [19]. The phosphorylation of Y904 and Y939 were induced following IL-2’s engagement of the receptor complex [19].

To determine whether additional phosphoregulatory JAK3 tyrosine residues are present within the enzyme, autokinase assays were coupled to mass spectrometry; this led to the detection of 10 novel JAK3 tyrosine autophosphorylation sites, including Y841 within the JH1 domain. Our multidisciplinary approach, combining molecular biology techniques with computational biophysics simulations, sought to determine a functional and structural role for autophosphorylated Y841 within the JAK3 JH1 kinase domain. Herein, evidence is provided that Y841 is a constitutively autophosphorylated site necessary for full STAT5 activation via IL-2R engagement, is associated with decreased pH sensitivity and the increased structural stability of the kinase domain, supports complementary JH1 kinase domain dimerization, increases structural stability, and is conserved across JAK family members and other kinases.

## 2. Results

### 2.1. Identification of Ten Novel JAK3 Tyrosine Phosphorylation Sites

**Figure 1:**
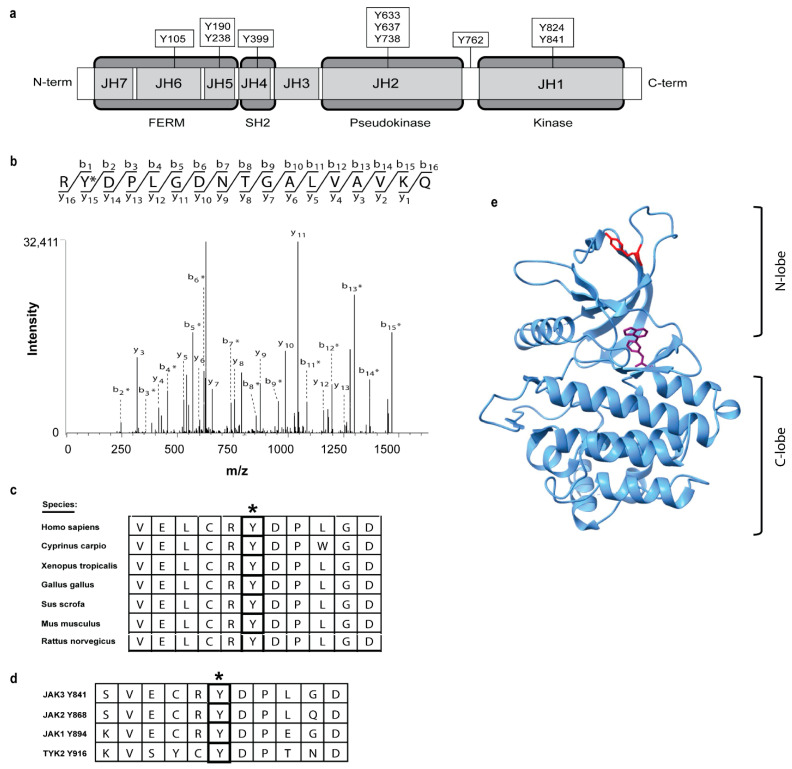
Ten novel human JAK3 tyrosine auto-phosphorylation sites identified via mass spectrometry. (**a**) Schematic model of 10 tyrosine phosphorylation sites identified following the JAK3 autokinase assay and their location within JAK functional domains. (**b**) Representative mass spectra for JAK3-Y841 (*) are shown for novel tyrosine phosphorylation sites. (**c**) Sequence alignment showing five amino acids up-stream and down-stream of JAK3-Y841 (*) across multiple species. (**d**) Partial sequence alignment for human JAK1 (Y894), JAK2 (Y868), and Tyk2 (Y916) aligned against JAK3-Y841 (*). (**e**) Ribbon structure of JAK3 JH1 domain (PDB 5TTV) depicting ATP (purple) and Y841 (red) within the kinase domain N-lobe.

To identify novel JAK3 phosphorylation sites, full-length human JAK3 cDNA was transfected into HEK293 cells for overexpression. Subsequently, protein was immunoprecipitated from HEK293 cells, subjected to an autokinase assay, separated via SDS-PAGE, and Coomassie stainable protein bands were excised for mass spectrometry (MS) analysis. Ten novel tyrosine autophosphorylation sites, including Y105, Y190, Y238, Y399, Y633, Y637, Y738, Y762, Y824, and Y841, were identified within JAK3 and are depicted in Figure 1a. Previously reported residues, including Y929 [20] and Y980 [21], were observed. Selected mass spectra data for the Y841 site are shown in Figure 1b. The remaining spectra for Y105, Y190, Y238, Y399, Y633, Y637, Y738, Y762, Y824, and Y929 are provided in Figure S1. The ten newly identified sites are all located within highly defined JAK functional domains, including the FERM, atypical SH2, pseudokinase and kinase domains (Figure 1a). Of particular interest, the kinase domain residue Y841 was observed to be strongly conserved throughout multiple species (Figure 1c), as well as all four JAK family members (Figure 1d). The strict evolutionary conservation of the residues surrounding Y841 includes hydrophilic residues (L838, V836, L844), charged amino acids (E837, R840, D842, D846) and a proximal proline (P843) residue. Moreover, Y841 is located within the N-lobe of the catalytic JH1 kinase domain (Figure 1e), separate from the ATP-binding pocket and center catalytic cleft.

### 2.2. JAK3 Kinase Domain Residues Y841 and Y929 Are Critical for Activation of Downstream Effector STAT5B

**Figure 2:**
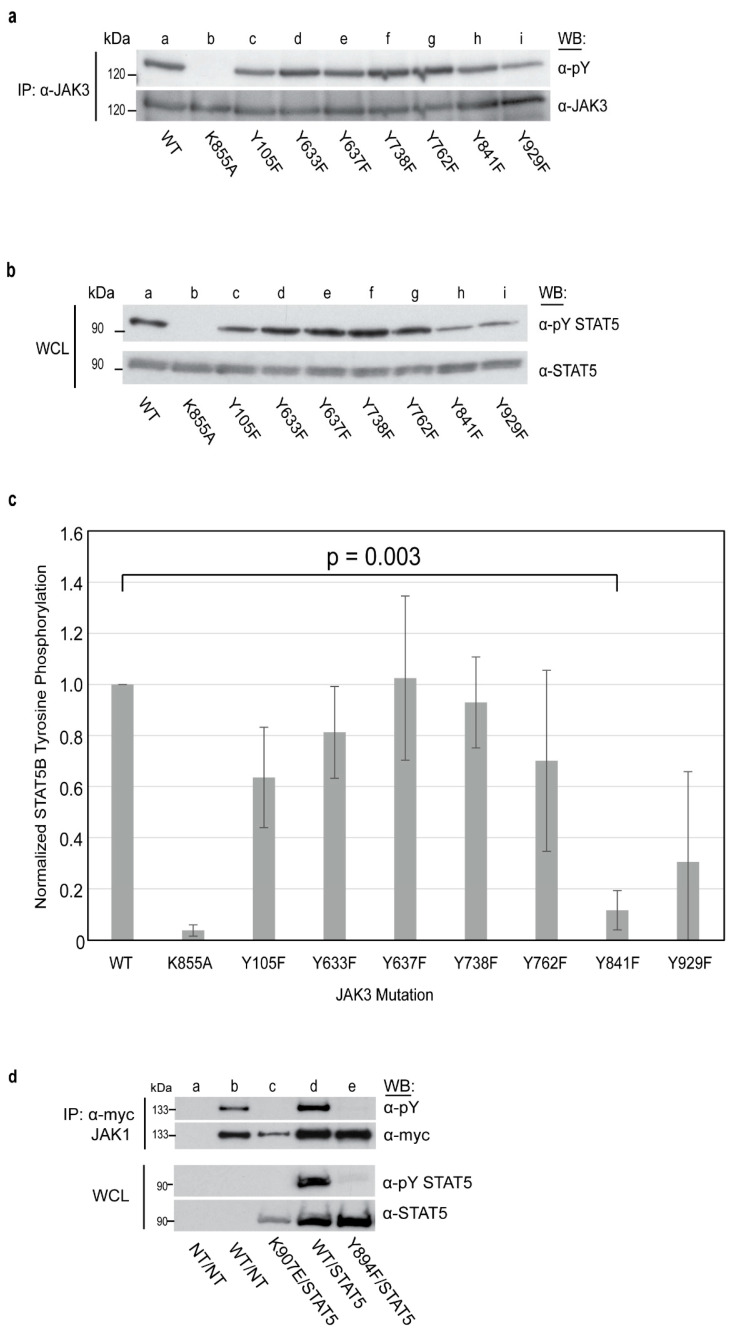
JAK3 Y841 and the homologous JAK1 Y894 are critical for the activation of STAT5B. (**a**) Hek293 cells were transfected with 10 μg WT (lane a) or mutant JAK3 plasmids (lanes b–i) and incubated for 48 h at 37 °C. Cells were harvested after 48 h, immunoprecipitated for JAK3 and Western blotted for total pY. Shown is a representative blot from n = 2 independent experiments. Individual points for replicate experiments are provided in Figure S2a. (**b**) Hek293 cells were transfected with 2 µg of WT (lane a) or mutant JAK3 plasmids (lanes b–i) with 2 µg of STAT5B plasmids (lanes b–i) and harvested after 48 h at 37 °C. Western blot was performed using anti-pYSTAT5 and reblotted for total STAT5 to confirm the equal protein expression and loading of STAT5B. Shown is a representative blot from n = 3 independent experiments. (**c**) Densitometry analysis using LI-COR Image Studio Digits was performed. Anti-pYSTAT5 signal was normalized against total STAT5 protein and graphed as the fold change in relation to WT JAK3. Error bars represent the mean +/− standard deviation from three independent experiments (n = 3). A paired Student’s *t*-test was performed between the densitometry values of the activated STAT5 from WT JAK3 and the individual JAK3 mutants. The individual data points for n = 3 replicate experiments and statistical analysis are provided in Figure S2b–d. (**d**) Hek293 cells not transfected (NT, lane a) or transfected with c-myc-tagged JAK1 WT (lanes b,d), kinase dead (K907E, lane c) or Y894F (lane e), and WT STAT5B (lanes c–e) was immunoprecipitated and Western blotted, as indicated with anti-pY or anti-myc. Whole cell lysates (WCL) obtained prior to immunoprecipitation were subjected to Western blot with anti-pY STAT5 or anti-STAT5, as indicated.

The impact of the novel phosphosites identified in this study within the FERM-SH2, pseudokinase and kinase domains on JAK3 enzymatic activity and downstream signal transduction was examined. For these studies, full-length JAK3 capable of interacting with signaling proteins such as STAT5 was favorable. JAK3 phospho-substitution mutations (Y to F) were generated for seven of the ten novel JAK3 phospho-sites, including the previously reported Y929 [20], and confirmed to be phosphorylated by this study. Phospho-substitution mutations were expressed in the mutated JAK3 protein for Y105, Y633, Y637, Y738, Y762, Y841 and Y929. Individual JAK3 tyrosine mutations, catalytically inactive JAK3 (K855A) or wild-type (WT) JAK3 cDNA were transfected into HEK293 cells, harvested 48 h later and JAK3 immunoprecipitated. The subsequent analysis did not require cytokine receptor stimulation, as JAK3 is highly concentrated within the cell and capable of autophosphorylation in the absence of cytokine stimulation [24,25,26]. Western blot analysis of the JAK3 protein using anti-pY and anti-JAK3 antibodies revealed a reduction in the total tyrosine autophosphorylation associated with the presence of phospho-substitution mutants within the JH1 kinase domain, Y841F and Y929F (Figure 2a, lanes h,i), in comparison to the WT protein (Figure 2a, lane a). Densitometry analysis and normalization against the WT protein confirmed that the total phosphotyrosine signal, a product of autophosphorylation, was reduced by an average of 49% and 61%, respectively (Figure S2a).

**Figure 3:**
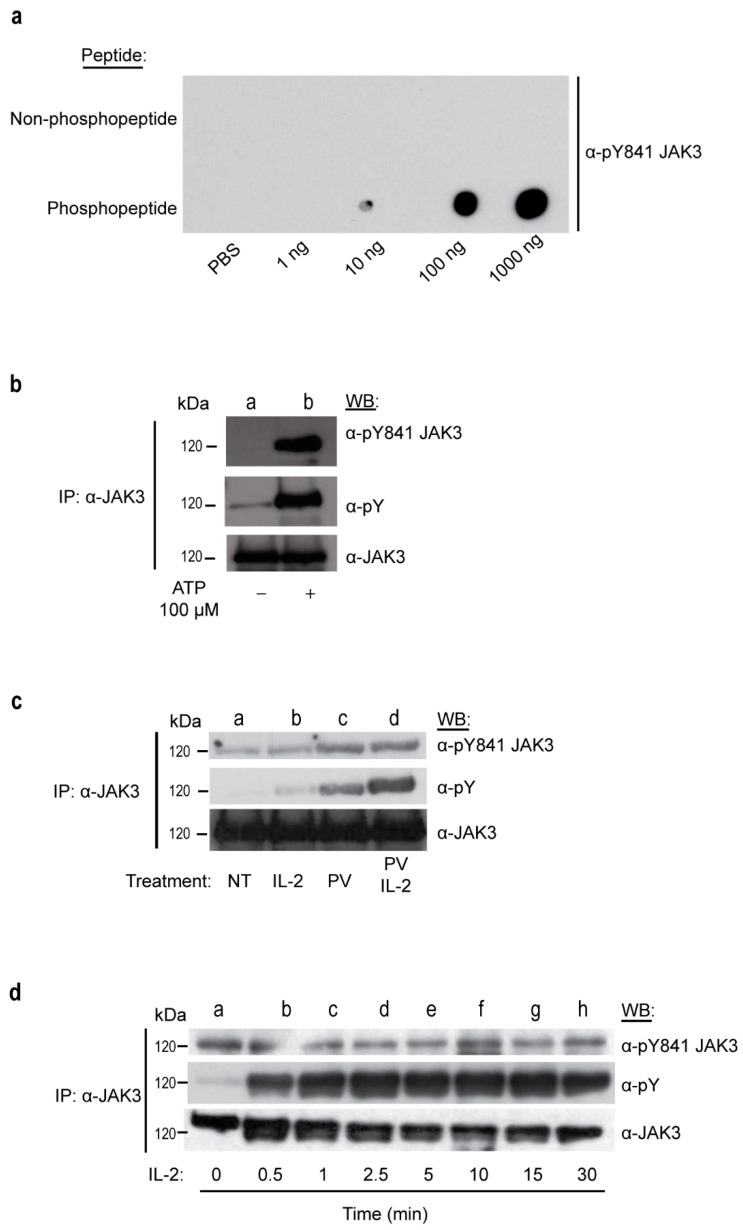
Phosphospecific monoclonal anti-pY841 JAK3 selectively detects phosphopeptide, recognizes auto-phosphorylated protein, and recognizes endogenous protein from pervanadate-treated YT cells. (**a**) A mouse monoclonal pY841 JAK3 antibody was tested against increasing concentrations (1 ng, 10 ng, 100 ng and 1000 ng) of non-phospho-Y841 peptide (top) vs. phospho-Y841 peptide (bottom). A representative blot from n = 2 independent experiments is shown. (**b**) In vitro kinase assay −/+ 100 µM of ATP (lane a,b) with immunoprecipitated JAK3 from transfected Hek293 cells. Western blot with anti-pY841 JAK3 antibody (top panel), total anti-pY antibody (middle panel) and reblot for JAK3 (bottom panel). (**c**) IL-2 stimulated YT cells without (lane a,b) or with (lane c,d) pervanadate (PV), and then immunoprecipitated for JAK3. Western blot with anti-pY841 JAK3, total anti-pY and reblotted for JAK3. (**d**) IL-2 stimulation time course of 0–30 min (lanes a–h) in YT cells, immunoprecipitated for JAK3 and probed for anti-pY841 JAK3; total pY and JAK3 reblot used to confirm equal protein loading. All Western blot data shown above (b–d) are representative blots from n = 3 independent experiments.

To separately assess the JAK3 activation of downstream STAT5, HEK293 cells were co-transfected with human STAT5B cDNA plus JAK3 phospho-substitution mutants (Y to F), catalytically inactive JAK3 (K855A), or WT JAK3 cDNA. Anti-pYSTAT5 was used as an indicator of activated STAT5 via Western blot. JAK3 mutants, namely Y841F and Y929F, drastically reduced STAT5B activation (Figure 2b, lanes h,i). A densitometry analysis of STAT5B activation by JAK3-Y841F showed a significant impact on kinase activity in comparison to cells transfected with WT JAK3 (*p* = 0.003, paired Student *t*-test) (Figure 2c and Figure S2c). The FERM domain mutation Y105F also resulted in reduced STAT5 activation, albeit lower than both Y841F or Y929F individually (Figure 2b, lane c, Figure 3c and Figure S2b). The individual data points of normalized STAT5B tyrosine phosphorylation and the statistical analysis are shared in Figure S2 for three independent replicates.

Because JAK3 and JAK1 form heterodimers when associated with common gamma-chain cytokine receptors [27], and because JAK3 Y841 is conserved within JAK family members, (Figure 1d), the JAK1 homologous residue Y984 phosphodeletion (Y to F) was created. The role of JAK1 Y894 was studied in HEK293 cells transfected with WT JAK1 or JAK1 Y894F and STAT5. Interestingly, the JAK1-Y894F protein failed to display a tyrosine autophosphorylation signal detectable by Western blot (Figure 2d, lane e). Similar to JAK3, JAK1-Y894F was unable to activate STAT5 (Figure 2d, lane e) and resembled the kinase dead JAK1 K907E mutation (Figure 2d, lane c).

### 2.3. JAK3 Y841 Represents a Constitutively Autophosphorylated Residue

To elucidate the function of phosphorylated Y841 (pY841) within JAK3, a monoclonal phospho-specific antibody was generated against the pY841-JAK3 peptide. Antibody specificity was determined by dot blot analysis using an immunizing phospho-peptide or nonphospho-peptide. Increasing amounts of peptide for site Y841 (1 ng–1000 ng) were spotted onto the PVDF membrane and immunoblotted using anti-pY841 JAK3. This newly generated monoclonal antibody recognized the phosphorylated peptide, but not the non-phosphorylated counterpart (Figure 3a). The pY841 JAK3 antibody was also used to Western blot the PVDF membrane spotted with non-phosphopeptide and phosphopeptide for JH2 residues (Y633, Y637, Y738) and JH1 sites (Y904 [19], Y929, Y939 [19]) in order to examine cross-reactivity. In all cases, the anti-pY841 antibody was not observed to detect any other JH2 or JH1 phosphopeptides within JAK3 (Figure S3).

The anti-pY841 JAK3 antibody was tested for its ability to recognize full-length phosphoprotein. First, HEK293 cells were transfected with wild-type (WT) human JAK3 cDNA. Immunoprecipitated JAK3 on sepharose beads was subjected to an in vitro autokinase assay in the absence or presence of ATP. The JAK3 protein was recognized by anti-pY841 JAK3 antibodies (Figure 3b, top panel), confirming that Y841 is autophosphorylated. Next, YT cells, which express endogenous JAK3, were pre-treated with the tyrosine phosphatase inhibitor pervanadate for 30 min to boost tyrosine phosphorylation and then stimulated with cytokine to activate endogenous JAK3. JAK3 was immunoprecipitated and Western blotted with the anti-pY841 JAK3 antibody. Tyrosine 841 was observed to be constitutively phosphorylated in IL-2 stimulated cells, despite making the cells quiescent via CO_2_ stripping and overnight culture in serum-free media (Figure 3c, top panel, lanes a–d). Protein isolated from pervanadate-treated cells exhibited a higher degree of Y841 phosphorylation (Figure 3c, lanes c,d). Lastly, JAK3 immunoprecipitated from non-pervanadate-treated YT cells stimulated with IL-2 from 30 sec to 30 min revealed basal levels of pY841 (Figure 3d, upper panel), regardless of the cytokine treatment duration; this is unlike the total phospho-tyrosine signal, which was cytokine-inducible (Figure 3d, middle panel).

### 2.4. JAK3 pY841 Is Constitutively Phosphorylated in Leukemic JAK3 A573V-Positive Cells and Various T-Cell Lines

**Figure 4:**
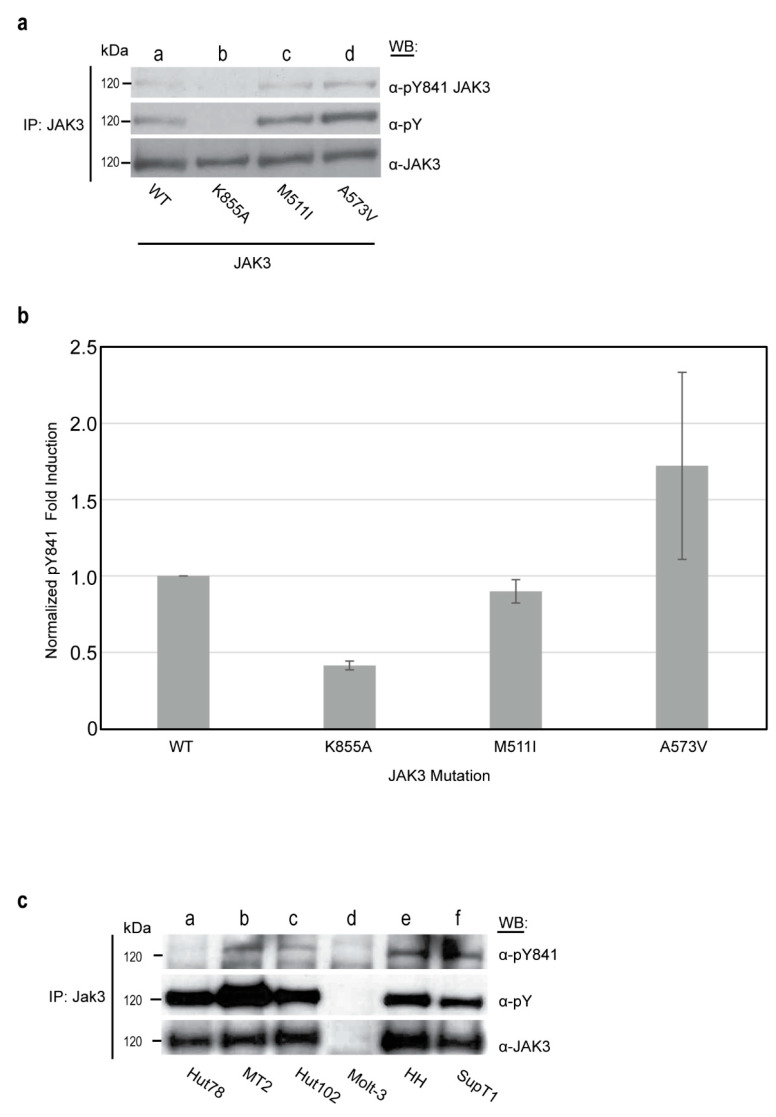
JAK3 pY841 is upregulated in cells transformed by the JAK3 A573V variant and various T-cell lines. (**a**) HEK293 cells transfected with WT (lane a), K855A (lane b), M511I (lane c) or A573V (lane d) JAK3 were immunoprecipitated for JAK3, separated by SDS-PAGE and transferred to the PVDF. Membranes were Western blotted using anti-pY841 JAK3, anti-pY and reblotted for total JAK3 protein to confirm equivalent loading. Representative data from n = 3 replicates shown. The individual data points for replicate experiments are available in Figure S4. (**b**) Densitometry analysis was performed using LI-COR Biosciences software. Anti-pY841 values were normalized against the total JAK3 protein and plotted as fold induction to WT JAK3. Error bars represent the mean +/− standard deviation from three independent experiments (n = 3). (**c**) The actively growing cell lines Hut78 (lane a), MT2 (lane b), Hut102 (lane c), Molt3 (lane d), HH (lane e), and SupT1 (lane f) were lysed and immunoprecipitated for endogenous JAK3. PVDF membranes were Western blotted with anti-pY841, anti-pY, or reblotted for total JAK3 to confirm the presence of protein. Representative data from n = 2 are shown.

Transforming variants of JAK3 are associated with various subtypes of hematopoietic malignancies; yet, the mechanisms of these transformations remain poorly understood [24,28,29,30,31]. To determine whether JAK3-Y841 is differentially phosphorylated in transforming JAK3 mutants, M511I and A573V, described previously by Martinez, et al. [24], were used. JAK3 WT or JAK3 K855A, M511I, or A573V mutant proteins were overexpressed in HEK293 cells, and immunoprecipitated JAK3 proteins were Western blotted with anti-pY841 JAK3 monoclonal antibodies. The JAK3 A573V variant protein displayed an average of a 1.7 fold increase in Y841 phosphorylation, while M511I was comparable to the WT protein (Figure 4a,b). Y841 was constitutively phosphorylated within A573V and M511I JAK3 variants similar to the WT protein.

The phosphorylation status of JAK3-Y841 in various human leukocyte lines, including Hut78, MT2, Hut102, MT2, Molt3, HH and SupT1 cells, was investigated. Hut78 (Figure 4c, lane a), MT2 (lane b), Hut102 (lane c), HH (lane e) and SupT1 (lane f) cells expressed sufficient levels of JAK3 protein to detect pY841-JAK3. Both Hut78 and Hut102 cells were derived from a 26-year-old T-cell lymphoma patient of African-American descent [32]. Interestingly, the virally transformed HTLV-1 T-cell leukemia/lymphoma cell lines MT2 and Hut102 displayed JAK3-Y841 phosphorylation, while the non-HTLV-1-positive parental cell line of Hut102, Hut78, did not (Figure 4c, lane a).

### 2.5. Phosphorylation of Y841 Decreases JAK3 JH1 Kinase Domain pH Sensitivity

**Figure 5:**
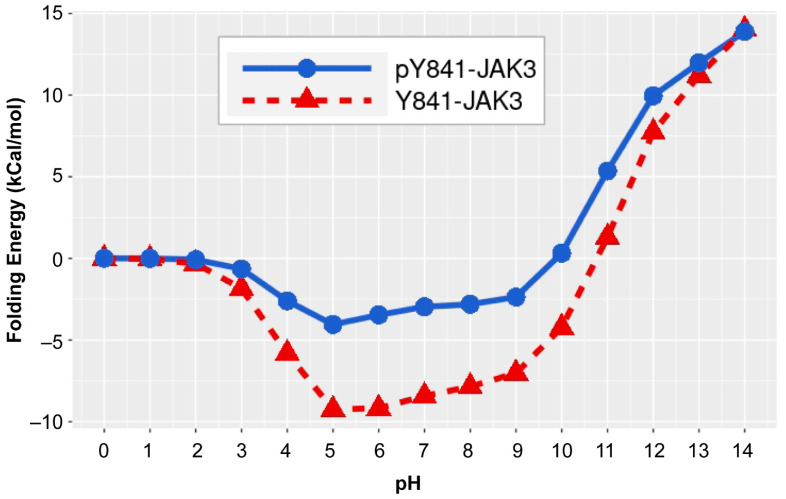
pH dependence of the folding energy for phosphorylated and non-phosphorylated Y841-JAK3. The folding energy values for non-phosphorylated Y841 (Y841-JAK3) and phosphorylated Y841 JAK3 (pY841-JAK3) were determined using PROPKA3, as shown by Equation (1) in Methods. Data points represent the difference between the kcal/mol values at the designated pH and the determined binding energy at pH = 0. A positive folding energy is indicative of a higher folding energy requirement compared to pH = 0, and negative values are indicative of a lower energy requirement compared to pH = 0.

To address the possible impact of Y841 phosphorylation on JAK3 folding, a pH dependence of folding energy analysis was performed. Non-phosphorylated and phosphorylated Y841 JAK3 states revealed a very similar trend of folding energy being present with increasing pH values (Figure 5). The most stable environment for both JH1 domain states occurred between pH 5 to 9. The JH1 kinase domain was less sensitive to pH changes (discernible by small changes in folding energy) when Y841 was phosphorylated compared to the non-phosphorylated Y841 protein. pY841 also stabilized the structure of the JH1 domain in both weak basic and acidic environments. The relatively stable folding energy of the non-phosphorylated Y841 JH1 domain was within the pH range of 5 to 9, while the pH of pY841 was between 4 to 9. The phosphorylation of Y841 decreased the pH sensitivity of the JAK3 kinase domain and enhanced its stability in different pH environments.

### 2.6. JH1 Kinase Domain Electrostatic Potential Is Altered by Y841 Phosphorylation to Favor Dimerization and Structural Stability

**Figure 6:**
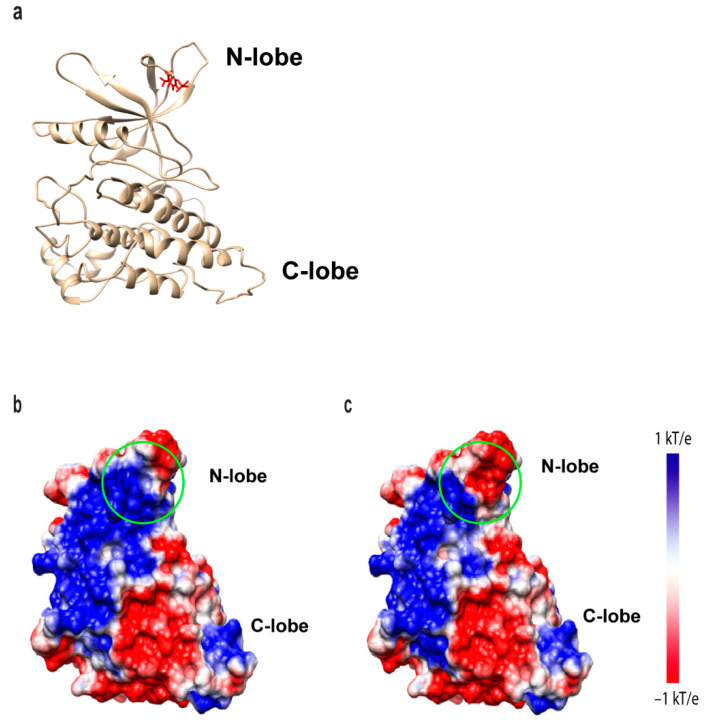
Structure of the JH1 kinase domain of JAK3 and surface electrostatic potential surrounding Y841. (**a**) View of the JAK3 JH1 kinase domain ribbon structure (amino acids 815-1124) is shown depicting Y841 (shown in red) located on the N-lobe. Positively and negatively charged surface regions are colored in blue (1 kT/e) and red (−1 kT/e), respectively. The Y841 surface region is circled in green for (**b**) non-phosphorylated Y841 kinase and (**c**) phosphorylated Y841 kinase.

Delphi and CHARMM36 were used to analyze the electrostatic potential of the JAK3 JH1 kinase domain, amino acids 815-1124 (Figure 6a), and revealed significant differences between the surface charge of the region surrounding Y841 in non-phosphorylated and phosphorylated forms. The positive electrostatic potential (blue) within the surrounding non-phosphorylated Y841 surface (Figure 6b) was predicted to be negatively charged (red) in the phosphorylated Y841 protein (Figure 6c).

**Figure 7:**
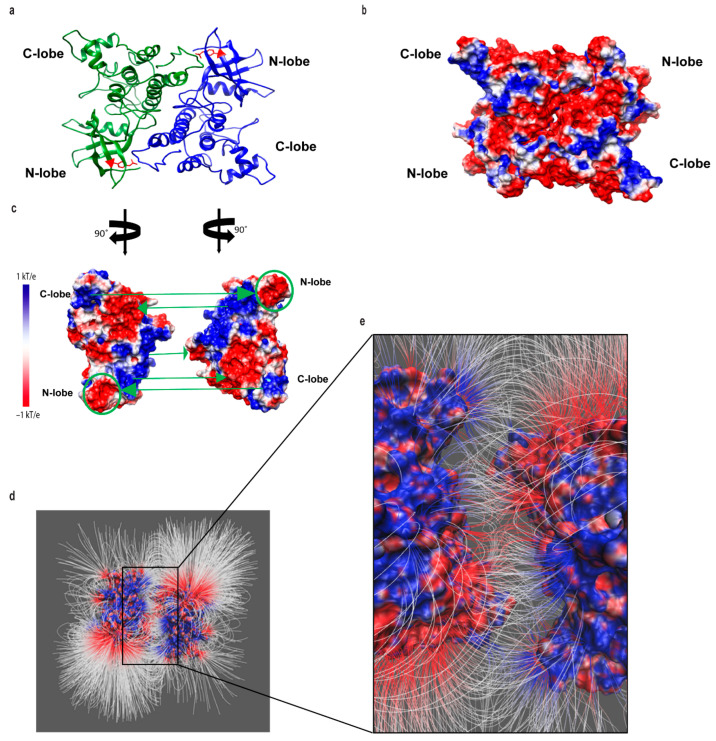
Dimerized structure of pY841 JAK3 determined using ZDOCK Server. (**a**) Structure of the pY841 kinase domain (amino acids 815-1124) dimer with Y841 depicted (red). (**b**) The electrostatic surface potential of the JH1 dimer depicted in (**a**). (**c**) The electrostatic surface potential for the binding interface of (**b**) opened by 90°. Green arrows depict binding pairs with the positive surface pointing to the corresponding negative surface of the dimer interface. Green circle encompasses the area containing the phosphorylated Y841 residue. (**d**) Depicted dimer in (**b**) was separated by 15 Å to show the electrostatic field lines between the interface. (**e**) Magnified image of kinase domain interface and electrostatic field lines in the cutout region.

To better understand how this change in the surface charge surrounding Y841 influences JH1 kinase domain juxtaposition, which is essential in JAK activation and signal transduction, the protein–protein docking of JAK3 amino acids 815-1124 was analyzed using the ZDOCK Server. From the top docking structures yielded, the structure with the largest binding surface area was selected and is shown in Figure 7a. The dimerized ribbon structure (Figure 7a) and corresponding electrostatic surface potential (Figure 7b) show geometrical alignment. Next, the electrostatic interactions between two JH1 monomers were studied by rotating open each monomer of the dimer in Figure 7a,b by 90° to reveal the binding interface. A complementary electrostatic surface potential was noticeable in the opened interfaces, revealing five potential binding pair interactions (Figure 7c, green arrows). Notably, the negatively charged pY841 region within one kinase domain N-lobe (Figure 7c, green circle) aligned to the positively charged C-lobe of the dimerized kinase. To validate this model of JH1 dimerization, the electric field lines depicted in Figure 7d,e illustrate strong interactions between two pY841 JAK3 kinase domains in this position and validate the stability of the predicted dimer structure. In Figure 7a, the intensive electrostatic connections (dense electrostatic field lines) depicted in Figure 7d,e are consistent with the five electrostatic complementary areas shown in Figure 7c. The JH1 dimer structure proposed in this study supports the role of pY841 in dimer formation.

## 3. Discussion

It is well established that the tyrosine phosphorylation of JAK3 is important for its signaling via the γc receptor and is required for proper T-cell functions [19,21,33,34]. Although 10 novel tyrosine autophosphorylation sites were reported in this study (Figure 1), only the two kinase domain residues impacted total JAK3 autophosphorylation and STAT5B tyrosine phosphorylation (Figure 2). The generation of a phospho-specific monoclonal antibody against JAK3-Y841 was highly selective for the phosphorylated form (Figure 3), allowing the residue to be further characterized. Subsequent studies determined this site to be constitutively phosphorylated, as assessed via autophosphorylation in vitro kinase assays, the isolation of JAK3 from T-cells treated with pervanadate, and endogenous protein from leukemic cell lines (Figure 3 and Figure 4). Herein, a computational biophysics analysis of the electrostatic surface potential and electrostatic field lines associated with pY841 suggests that phosphorylation at this site is energetically preferred to form a complementary JH1 dimer interface and to stabilize an active structure (Figure 5, Figure 6 and Figure 7). Previously reported molecular dynamic simulations of pY841 within the JAK3 kinase domain further support its role in activation. Recently, Sun et al. showed that pY841 increases the elasticity of the enzymatic cleft between N- and C-lobes, promotes the binding of ATP, and may initiate the separation of JH1–JH2 from the inactivated to activated position [35], enabling two JH1 dimers to form, as described here (Figure 7). Given the number of autophosphorylated sites identified in this study, it is likely that the Y841 region is not the sole area controlling structural stability and dimer formation. This topic is highly interesting and is being investigated.

Given its function and location, we believe that this site also serves a role in stabilizing the JAK3 surface, allowing molecules, such as STATs, to bind, become activated and achieve maximum capacity for propagating the cell signal transduction event. While the full-length human JAK3 crystal structure has not been established, recently, Glassman et al. reported the complexation of the full-length mouse JAK1-activated homodimer with the partial cytokine receptor structure [36]. The alignment of the Glassman mouse JAK1 structure with our I-Tasser-determined model of JAK3 showed a high degree of alignment along the JAK3 FERM-SH2-JH2 regions, imparting confidence in our model. The most drastic difference was seen within the JH1 domain, likely a result of the different states that the JH1 domain can form when bound to ADP, ATP, the phosphorylation state, the activation state, or via dimerization. Additionally, the Glassman JH1 domain mouse structure is not bound to the cytokine receptor and thus is not a complete portrayal of the juxtaposed kinase domain. Future work will seek to fully establish these JH1 formations.

Past cancer studies investigated the transforming role of JAK3 mutations and their capacity to disrupt aberrant activation using selective inhibitors such as CP-690,550 [24,37,38,39]. Ongoing modeling studies of human JAK3, based on the Glassman et al. structure, seek to understand how leukemia and lymphoma mutations disrupt normal function. Positionally, A573V is located in the analogous JH2 nucleotide-binding pocket, while M511I is found within the SH2-JH2 loop. Although transforming the mutations cluster within the JH2 domain [40,41], their pathogeneic attributes may not be limited to a single mechanism of dysregulation. The differential detection of the pY841 signal here among transforming mutations supports this hypothesis. The detection of pY841 within A573V JAK3 (Figure 4a,b) and in various T-cell leukemia/lymphoma cell lines (Figure 4c) suggests that this site might serve as a unique biomarker for hyperactive kinase activity. Increased stabilization associated with the phosphorylation of Y841 (Figure 5, Figure 6 and Figure 7) may enable an increase in STAT and the activation of other downstream molecules in the absence of cytokine, therefore promoting the dysregulation of the tightly controlled signaling cascade associated with oncogenesis [42,43]. Ongoing studies seek to probe human patient tumor samples in order to determine whether this site is constitutively phosphorylated in primary hematopoietic tumors.

**Figure 8:**
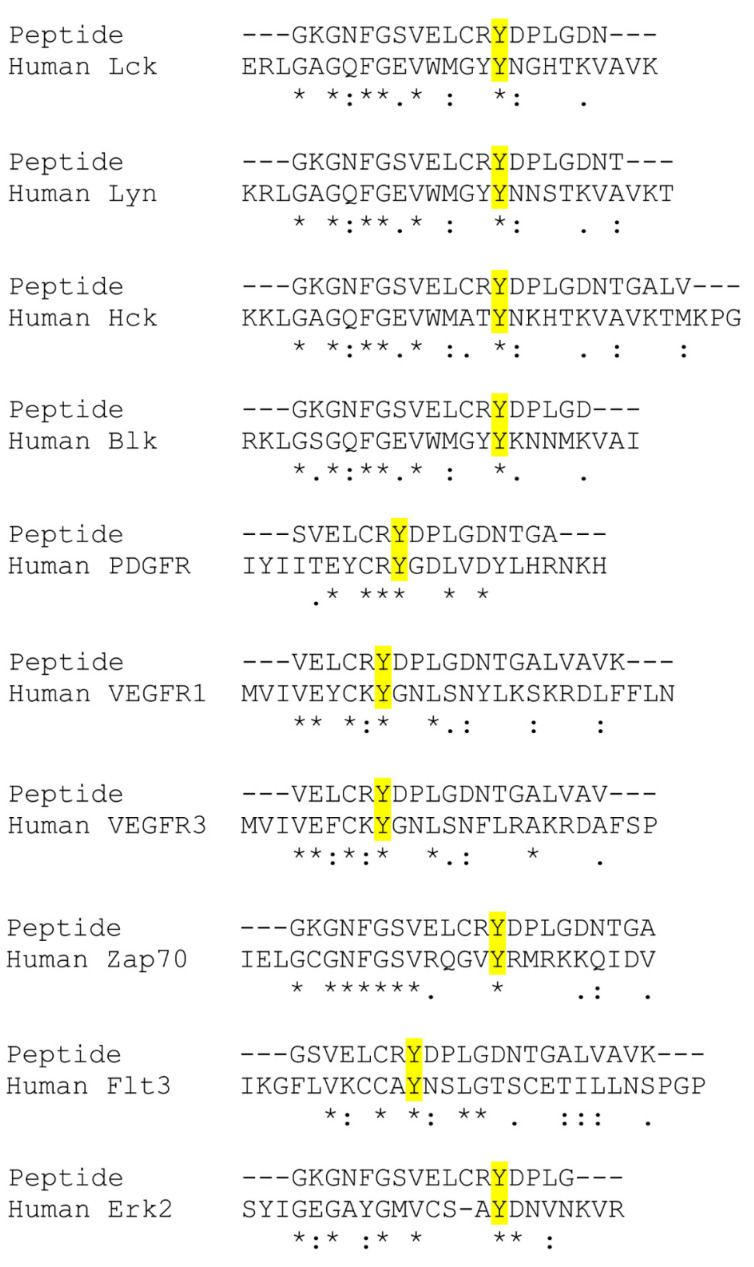
Partial sequence alignments for tyrosine and serine/threonine kinases found to possess a homologous Y residue at the positionally conserved JAK3 Y841 site. The partial JAK3 sequence (qlgkgnfgsvelcrYdplgdntgalvavk) containing Y841 (centered) was aligned with the full-length sequence for 95 kinases from the human kinome using the ClustalW2 Pairwise Sequence Alignment Tool (EMBL-EBI). Shown are the partial alignments for the JAK3 peptide containing Y841 aligned with homologous regions of human Lck (Y264), Lyn (Y267), Hck (Y282), Blk (Y260), Zap70 (Y231), PDGFR-B (Y686), VEGFR1 (Y876), VEGFR3 (Y1009), Flt3 (Y498), and ERK2 (Y43). Homologous Y residues are highlighted within individual kinase alignments. (*) indicates fully conserved residues, (:) indicates conservation between groups of strongly similar properties, and (.) indicates conservation between groups of weakly similar properties.

Ultimately, Y841 might play a greater functional role in kinases other than only JAKs, which share a highly conserved sequence homology localized to JAK3-Y841 (Figure 1d). The findings from this study can be used to better understand the role of tyrosine phosphorylation in JAK and other kinase-driven diseases. Indeed, a limited BLAST search revealed that JAK3 Y841 is positionally conserved in many tyrosine and serine/threonine kinases within the human kinome (Figure 8). JAK3-Y841 is notably conserved in the Src family members Lck, Hck, Blk, and Lyn, the growth factor receptors PDGFR, VEGFR1 and VEGFR3, the oncogenes Flt3 and c-Kit, Zap70 and the serine/threonine kinase Erk2. Several studies have previously identified conserved regions of the catalytic domain shared by hundreds of kinases that are necessary for peptide binding, catalysis, phospho-transfer and ATP binding [44,45,46]. The newly identified JAK3-Y841 represents a potentially novel mechanism that promotes the phosphorylation of optimal substrates by kinases. Moreover, this study’s computational findings related to JAK3-Y841 phosphorylation (Figure 5, Figure 6 and Figure 7) strongly suggest that this conserved tyrosine residue is important in stabilizing the kinase domain structure within JAKs and other kinases. It is conceivable that this site acts as a universal regulator in kinases across multiple families and may therefore serve as a novel therapeutic target for the development of inhibitors useful in the treatment of various kinase-driven cancers and other human pathologies.

## 4. Materials and Methods

### 4.1. Cell Culture and Treatments

The human natural killer (NK)-like cell line YT [47], the T-cell lines MT2 [48], Hut78 [32,49], Hut102 [32,49], HH (ATCC), Molt3 (ATCC), and SupT1 (ATCC), in addition to the human embryonic kidney cell line HEK293 (ATCC), were maintained in complete media (RPMI 1640 (Thermo Scientific Inc., Waltham, MA, USA) using 10% fetal bovine serum (FBS; Atlanta Biologicals), 2 mM of L-glutamine (Cellgro, Corning, NY, USA), and penicillin–streptomycin (50 IU/mL and 50 μg/mL, respectively; Cellgro) at 37 °C with 5% CO_2_. All cells were made quiescent by growing them to exhaustion (concentrations >5 × 10^5^ cells/mL) prior to stimulation with 10,000 IU of human recombinant IL-2 (NCI Preclinical Repository, Bethesda, MD, USA) for the indicated times. Treatments were performed at 37 °C using 1 × 10^8^ cells per treatment. Unstimulated cells were used as control samples. For pervanadate treatments, 100X pervanadate was prepared as previously described and used at a final concentration of 2.5 mM [50]. Pervanadate-treated cells (1 × 10^8^ cells/mL) were incubated for 30 min at 37 °C.

### 4.2. Transfections

For the JAK3/STAT5 activation studies, HEK293 cells were transfected with 2 µg of pcDNA3.1/human JAK3 plasmid [19] alone or with 2 µg of STAT5B plasmid (OriGene, Rockville, MD, USA) per confluent in 10 cm dishes. Cells were harvested 48 h post transfection. Transient transfections of HEK293 cells were performed using Lipofectamine 2000 (Invitrogen, Waltham, MA, USA) according to the manufacturer’s instructions. For JAK3 in vitro kinase studies, HEK293 cells were transfected with 2 µg of plasmid pcDNA3.1/human JAK3 [19] per confluent in 10 cm dishes. Cells were harvested 48 h post transfection.

### 4.3. Identification of Ten Novel JAK3 Tyrosine Phosphorylation Sites via Mass Spectrometry

For these assays, cells were immunoprecipitated for JAK3 and then subjected to an autokinase assay [25] coupled to microcapillary liquid chromatography–tandem mass spectrometry (LC–MS/MS), which was performed by the Taplin Mass Spectrometry Facility (Harvard University) using methods designed for phosphorylation analysis. The phosphorylation sites were compared using unactivated (−ATP) and activated JAK3 (+ATP) autokinase assays in order to identify inducible phosphorylation events, following the Taplin Mass Spectrometry methods, as follows. Excised protein gel bands were reduced and then alkylated prior to in-gel trypsin digestion [51]. After being washed and dehydrated, the gel pieces were rehydrated with 50 mM of ammonium bicarbonate solution containing 12.5 ng/µL of modified sequencing-grade trypsin (Promega, Madison, WI, USA) at 4 °C, prior to being stored at 37 °C overnight. Peptides were extracted and dried via speed-vac (1 h), and stored at 4 °C until analysis. Reconstituted samples (5–10 µL of 2.5% acetonitrile, 0.1% formic acid) were loaded onto a nano-scale reverse-phase HPLC capillary column created by packing 2.6 µm of C18 spherical silica beads into a fused silica capillary (100 µm inner diameter x ~30 cm length) with a flame drawn tip [52] via a Famos autosampler (LC Packings, Waddinxveen, Netherlands). A gradient was formed and peptides were eluted with increasing concentrations of 97.5% acetonitrile and 0.1% formic acid. The eluted peptides were subjected to electrospray ionization and entered into a LTQ Orbitrap Velos Pro ion trap mass spectrometer in order to detect, isolate and fragment each peptide. The identities of the proteins were determined by matching the peptides with the acquired fragmentation pattern using Sequest (ThermoFinnigan, San Jose, CA, USA) [53]. The phosphorylation of serine, threonine, or tyrosine (79.9663 mass units) was included in database searches in order to determine the phosphopeptides and phosphorylation assignments, which was performed using the Ascore algorithm [54].

### 4.4. Generation of Monoclonal Anti-pY841 Antibodies

Phospho-specific monoclonal antibodies were generated against Y841 via GenScript Inc. (Piscataway, NJ, USA). Briefly, the phosphopeptide (KGSVELCRpYDPLGDNT) corresponding to JAK3 pY841 was conjugated with KLH as an immunogen and BALB/c mice were immunized. All mice showed a satisfactory immune response and the optimal mice were used for cell fuion and hybridoma production. Hybridoma cells were produced and grown to a 1L volume in DMEM plus 10% FBS media until confluency was reached. The monoclonal antibodies produced in FEM plus low-IgG FBS and secreted by these hybridoma cells into the supernatant were purified using a Protein A affinity column, followed by elution with 0.1M citric acid (pH 3.5).

### 4.5. Dot Blot Analysis

Phosphopeptides (KGSVELCRpYDPLGDNT) and non-phosphopeptides (KGSVELCRYDPLGDNT) were diluted in water and spotted at increasing concentrations onto methanol-activated PVDF membranes. The membranes were allowed to dry, re-activated using methanol and then incubated with 1% BSA blocking buffer (20 mM Tris, 137 mM NaCl, 0.25% Tween-20, 1% Bovine Serum Albumin (BSA)) for 1 h at room temperature, followed by incubation with the newly generated phosphospecific monoclonal antibody anti-pY841 JAK3 (GenScript, 1:5000), as described previously [55].

### 4.6. Solubilization of Proteins, Immunoprecipitation and Western Blot

Cells were pelleted and solubilized in lysis buffer (10 mM of Tris-HCl (pH 7.6), 5 mM of EDTA (pH 8.0), 50 mM of NaCl, 30 mM of Na_4_P_2_O_7_, 50 mM of NaF, 1 mM of Na_3_VO_4_, 1% Triton X-100) containing 1 mM of phenylmethylsulfonyl fluoride (PMSF), 5 µg/mL of aprotinin, 2 µg/mL of leupeptin, and 1 µg/mL of pepstatin A, and rotated end-over-end at 4 °C for 1 h [25]. Whole-cell lysates were clarified via centrifugation (15,000× *g*, 15 min, 4 °C). For immunoprecipitation (IP) reactions, supernatants were rotated with 5 µg of anti-JAK3 rabbit polyclonal antibody [56] for Kit225, YT, MT2, Hut78, Hut102, requiring 1 × 10^8^ cells per IP, 3 μg (transfected HEK293 cells) of anti-Myc mouse monoclonal (sc-40, 9E10; Santa Cruz, Dallas, TX, USA) or 3 µg of anti-JAK3 rabbit polyclonal antibody [56] for the remaining cell lines for 2 h at 4 °C. Immune complexes were captured via incubation using Protein A-Sepharose beads (Rockland Immunochemicals, Pottstown, PA, USA), which were rotated for 1 h at 4 °C. The beads were then washed three times with ice cold lysis buffer and eluted by boiling them for 5 min in 2X SDS sample buffer (50 mM of Tris-HCl (pH 6.8), 100 mM dithiothreitol, 2% SDS, 0.02% bromophenol blue, 10% glycerol, pH 6.8). Samples were resolved using 7.5% SDS-PAGE and transferred to a polyvinyl difluoride (PVDF) membrane, which was subsequently blocked with 1% BSA blocking buffer (described above) for 1 h at room temperature. Western blot analysis was performed by incubating the membrane with an anti-pY841 JAK3 monoclonal antibody (1:1000), anti-pY antibody (clone 4G10, 1:1000; MilliporeSigma, Burlington, MA, USA) and anti-pY694 STAT5 (#9351, 1:1000; Cell Signaling, Danvers, MA, USA) antibody overnight at 4 °C, or anti-JAK3 (ab45141, 1:1000; Cambridge, United Kingdom), anti-STAT5 (C-17, 1:1000; Dallas, TX, USA) or anti-Myc (sc-40, 1:1000; Dallas, TX, USA) for 1 h at room temperature. Assays were developed using horseradish peroxidase-conjugated goat anti-mouse or anti-rabbit IgG (heavy plus light chains, 1:5000; SeraCare, Milford, MA, USA), and visualized via enhanced chemiluminescence and X-ray film. Individual Western blots were normalized by reblotting the PVDF membranes in order to determine the total protein, as described previously [25]. Western blot signals were quantified via densitometric analysis using Image Studio Digits Version 5.2.5 (LICOR, Lincoln, NE, USA).

### 4.7. Autokinase Assay

HEK293 cells were transfected using the appropriate expression vectors for JAK1 (RC213878; OriGene, Rockville, MD, USA) or JAK3 [19] prior to lysis with the Triton lysis buffer described above. JAK proteins were immunoprecipitated using anti-JAK3 or anti-Myc antibodies and captured using Protein A-Sepharose, as described above. The beads were washed three times with cold lysis buffer and once with ice cold kinase buffer (25 mM of HEPES (pH 7.3), 1% Triton X-100, 100 mM of NaCl, 10 mM of MgCl_2_, 3mM of MnCl_2_ and 50 µM of sodium orthovanadate). The kinase reaction was initiated via the addition of 100 µM of ATP, and then the beads were incubated at 37 °C for 20 min. The reactions were quenched by washing the Protein A-Sepharose beads with lysis buffer and eluting the material using 2X SDS sample buffer (described above). The samples were resolved using 7.5% SDS-PAGE, and the tyrosine phosphorylation levels of JAK3 were assessed via Western blotting using the anti-pY841 JAK3 monoclonal antibody, anti-pY antibody (Millipore), or anti-JAK3 (Abcam) and anti-myc (Santa Cruz) for 1 h at room temperature. The assays were developed using horseradish peroxidase-conjugated goat anti-mouse or anti-rabbit IgG (heavy plus light chains; SeraCare), and visualized by using enhanced chemiluminescence and X-ray film.

### 4.8. Relative Folding Energy Calculation

The net charge of the unfolded and folded JAK3 states was calculated using PROPKA3 [57,58] (see Table S1), between pH values of 0 to 14. Charge–charge interactions between the residues of the unfolded state caused shifts in the pKa values [59], which in turn affected the pH dependence of the folding stability, which determined using the following equation [60]:

(1) $$\Delta G\left(pH\right)=RTln10\int_{pH_{0}}^{pH}{(Q}_{f}-Q_{u})dpH$$

where *Q_f_* and *Q_u_* stand for the net charge of the folded and unfolded states, respectively. *R* is the universal gas constant value of 1.9872 × 10^−3^ kcal/(Mol*K), and *T* is the temperature, which was set to 300 K.

### 4.9. Structural Modeling of JAK3

The full-length JAK3 structure was modeled using I-TASSER [61]. The JH1 kinase domain (aa 815 to 1124) was extracted from the full-length JAK3 model for electrostatic analysis, molecular dynamics simulations and docking. The JAK3 JH1 I-TASSER structure showed a high degree of alignment with the experimentally determined PDB 5TTV structure and depicted segments that were not visible via PDB 5TTV (Figure S5). The phosphorylation of Y841 (pY841) was built using CHARMM-GUI [62].

### 4.10. Molecular Dynamic Simulation

The phosphorylation of Y841 and the solvation box were set up using CHARM-GUI, with the force field of JAK3 Y841 and pY841 using CHARMM36 [63] via NAMD-2.12 [64]. The system was solvated using typeTIP3P [65] water, and 150 mM of KCl was used to ionize the system. A periodic boundary condition was applied to the simulating box, and particle mesh Ewald was used for long-range electrostatic interactions. The simulation was carried out in two steps. The first step was equilibration using NPT (Isothermal-isobaric model). The set parameters for the equilibration step included a temperature of 310 K and a pressure of 1 atm, using a Langevin thermostat with a damping coefficient of 1/ps and a Nosé–Hoover Langevin piston barostat with a decay period of 25 fs, respectively. The temperature was reassigned every 500 steps. In the course of the equilibration step, a constraint was applied to JAK3. During the second step, production run, the NPT ensemble was continued for 100 ns (Figure S6). The constraints on JAK3 were released during the production run.

### 4.11. Electrostatic Calculation

The electrostatic features of JAK3 were calculated using Delphi [66]. The charge and radius of the atoms were calculated using CAHRMM36 [63] and assigned using PDB2PQR [67]. The dielectric constants were set as 2 and 80 for JAK3 and water, respectively. The salt concentration was set as 0.15 M. The probe radius, filling ratio of protein, and resolution were set to 1.4 Å, 0.70, and 2 grids/Å, respectively. The electrostatic potential on the surfaces was visualized using Chimera [68] and VMD [68].

### 4.12. JAK3 JH1 Kinase Domain Dimer Docking

Two monomers of the JAK3 JH1 kinase domain phosphorylated at Y841 were used for docking via the ZDOCK sever [69]. Based on the top 10 predictions suggested by ZDOCK, the dimer structure with the largest binding area (Minimal Accessible Surface Area (ASA) of the dimer complex) was chosen for further analysis. Additionally, to better view the binding of the two JH1 monomers, each monomer was rotated 90° to show their electrostatic potential and also separated by 15 Å to show their electric field lines [70].

## Data Availability

Not applicable.

## Supplementary Materials

The following supporting information can be downloaded at: https://www.mdpi.com/article/10.3390/ijms241511928/s1.
