# Supplementary material for: JAK3 Y841 Autophosphorylation Is Critical for STAT5B Activation, Kinase Domain Stability and Dimer Formation
Source: Int J Mol Sci. 2023 Jul 25;24(15):11928. doi: 10.3390/ijms241511928 (PMC10418363; doi:10.3390/ijms241511928)
Supplement: Supplementary file 1 [file ijms-24-11928-s001.zip › ijms-2455767-supplementary.pdf]

Figure S1

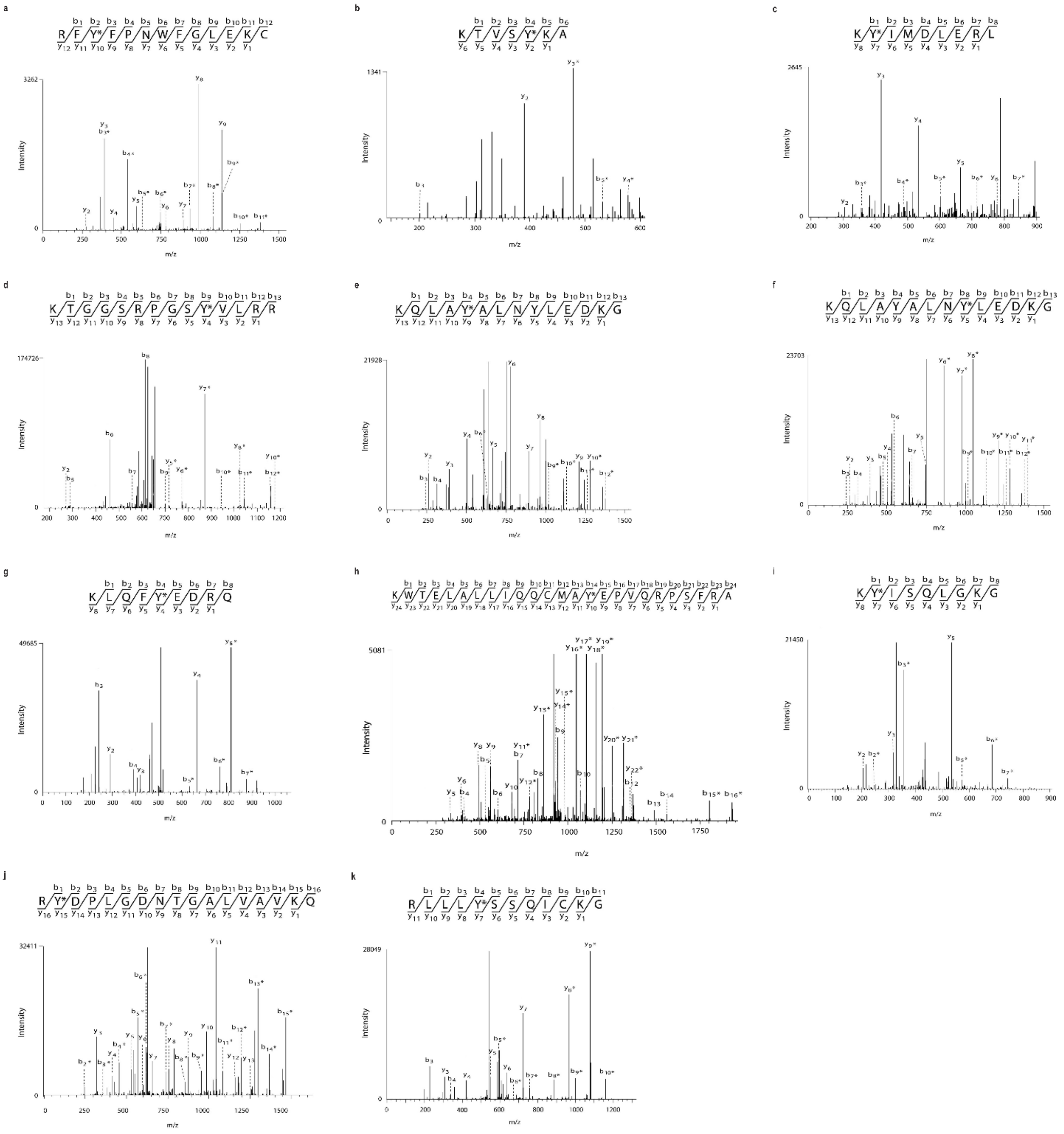

Figure S1. Mass spectra of identified JAK3 tyrosine phosphorylation sites. Mass spectra are shown for JAK3 tyrosine phosphorylation revealed by mass spectrometry analysis from JAK3 autokinase assays. (a) Mass spectrum of pY105. (b) Mass spectrum of pY190. (c) Mass spectrum of pY238. (d) Mass spectrum of pY399. € Mass spectrum of pY633. (f) Mass spectrum of pY637. (g) Mass spectrum of pY738. (h) Mass spectrum of pY762. (i) Mass spectrum of pY824. (j) Mass spectrum of pY841. (k) Mass spectrum of pY929.

Figure S2

a

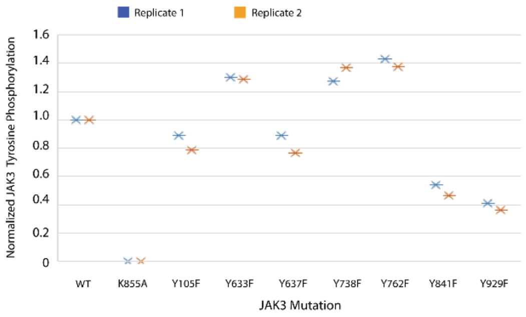

b

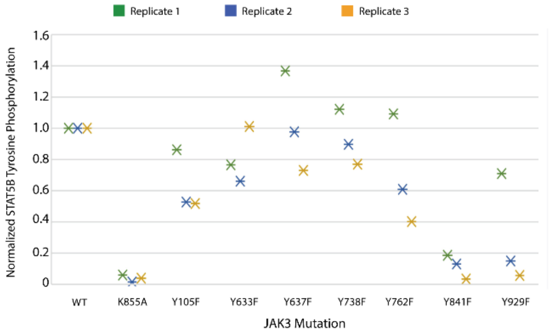

c

Pair	Student t-test
WT and K855A	0.000174
WT and Y105F	0.084878
WT and Y633F	0.212279
WT and Y637F	0.905769
WT and Y738F	0.564627
WT and Y762F	0.281038
WT and Y841F	0.002519
WT and Y929F	0.076489

d

Class Level Information

Class	*Levels*	*Values*
Mutant	9	K855A WT Y105F Y633F Y637F Y738F Y762F Y841F Y929F

Source	*DF*	*Sum of Squares*	*Mean Square*	*F Value*	*Pr > F*
Model	8	3.41808953	0.42726119	8.31	0.0001
Error	18	0.92507239	0.05139291		
Corrected Total	26	4.34316192			

R-Square	*Coeff Var*	*Root MSE*	*Y Mean*
0.787005	36.67235	0.226700	0.618177

Source	*DF*	*Anova SS*	*Mean Square*	*F Value*	*Pr > F*
Mutant	8	3.41808953	0.42726119	8.31	0.0001

Figure S2. Statistical analysis of Normalized JAK3/STAT5B Tyrosine Phosphorylation. (**a**) Individual points for n = 2 replicates used for Figure 2a are displayed. (**b**) Individual points for n = 3 replicates used for Figure 2c are displayed. (**c**) Paired student t-test comparison between wild-type (WT) JAK3 protein and individual JAK3 single amino acid mutations of tyrosine (Y) to phenylalanine (F). JAK3 K855A is kinase dead protein. (**d**) One-way Anova multiple comparison for STAT5B tyrosine phosphorylation associated with JAK3 mutants and WT protein. The null hypothesis of the one-way Anova is “H0: all 9 mutants have the same mean”. H0 was rejected by One-way Anova.

Figure S3

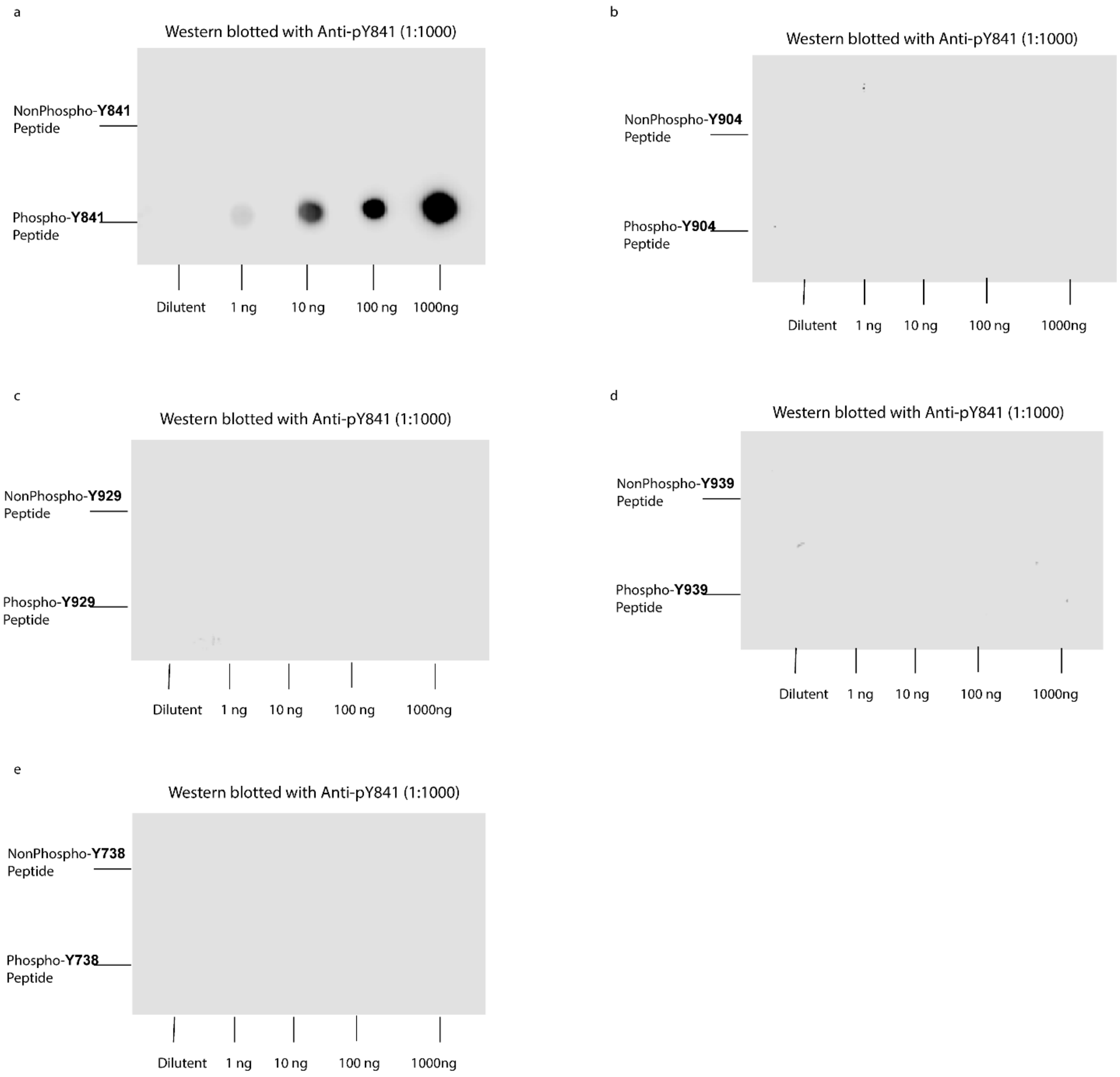

Figure S3. Anti-pY841 selectively recognizes pY841-JAK3 and not other pseudokinase or kinase phospho-tyrosine residues. Dot blot of JAK3 non-phosphopeptide and phospho-peptides for (a) JAK3-Y841 (KGSVELCRYDPLGDNT) (b) JAK3-Y904 (CLVMEYLPSG) (c) JAK3-Y929 (CKDASRLLLYSSQI) (d) JAK3-Y939 (CKGMEYLGSR) and (e) JAK3-Y738 (AKKLQFYEDRQQL) using pY841-JAK3 antibody. Non-phosphopeptide and phosphopeptide were spotted at increasing concentrations from 1 ng to 1000 ng onto PVDF membrane prior to Western blot analysis. Coomassie stain was used to verify the spotting of peptide onto membrane. 1000 ng spots of non-phosphopeptide and phosphopetides were detected equally on all membranes shown.

Figure S4

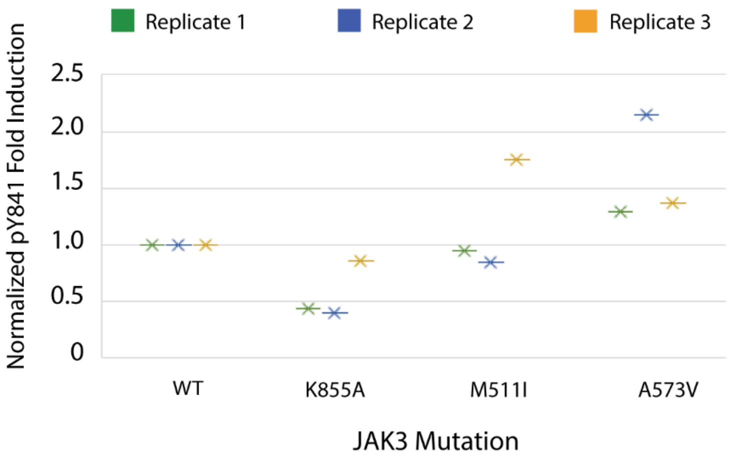

Figure S4. Individual points for Normalized Y841 JAK3 Phosphorylation. The depicted data points from n=3 replicate experiments used for Figure 4b are presented here.

Table S1

pH	Unfolded (before Phos)	Folded (before Phos)	Unfolded (after Phos)	Folded (after Phos)
0	36.00	35.94	36.00	35.98
1	35.97	35.48	35.97	35.85
2	35.67	33.12	35.67	34.72
3	33.00	26.33	33.00	29.72
4	20.29	14.55	20.29	17.86
5	3.29	3.41	3.29	4.28
6	-2.24	-0.98	-2.24	-1.40
7	-3.14	-2.18	-3.14	-2.89
8	-4.65	-3.27	-4.65	-3.90
9	-10.87	-6.17	-10.87	-6.37
10	-22.95	-13.70	-22.95	-14.50
11	-35.62	-24.83	-35.62	-27.89
12	-44.03	-38.19	-44.03	-40.65
13	-56.42	-51.69	-56.42	-53.19
14	-61.29	-58.69	-61.29	-58.94

Table S1. Net charge of JAK3 states calculated using PROPKA3 for pH 0 to 14.

Figure S5

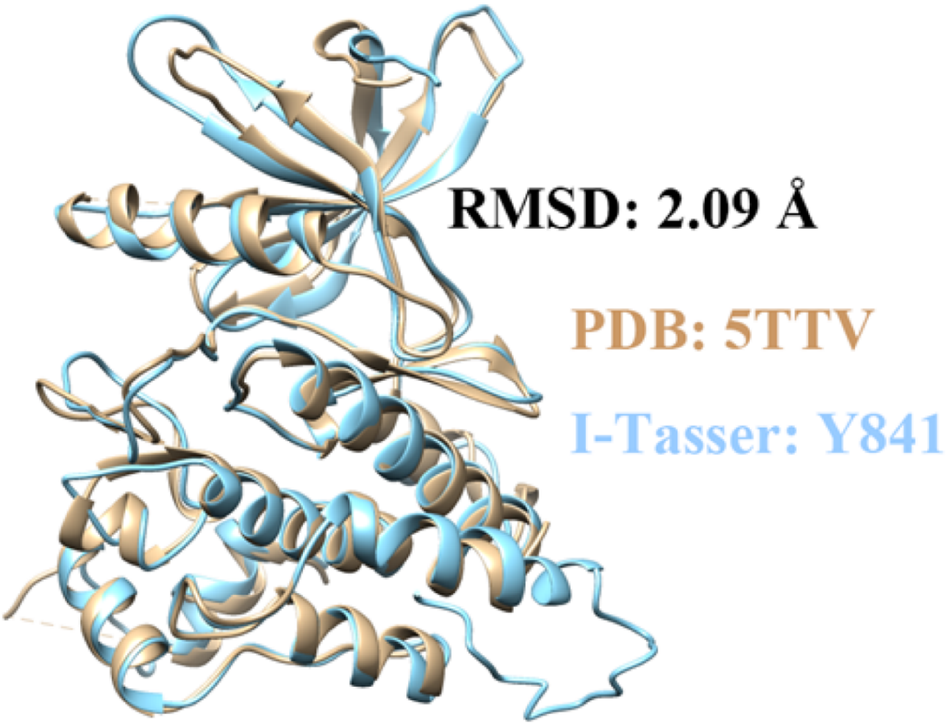

Figure S5. Alignment of PDB 5TTV and I-Tasser JAK3 Structures. A comparison of the aligned structures shows portions of the JAK3 structure not captured by PDB 5TTV and provided by I-Tasser.

Figure S6

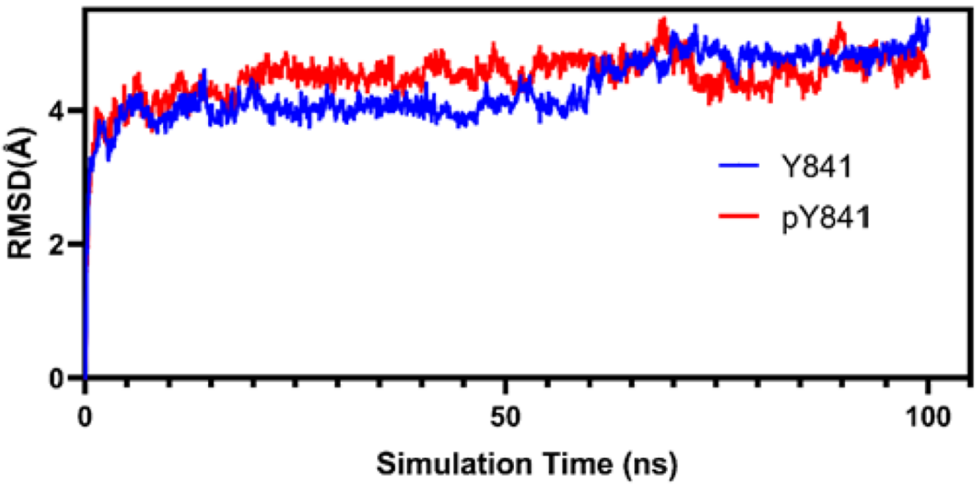

Figure S6. RMSD of Kinase in 100 ns Stimulation. Second step of molecular dynamic simulation, production run, NPT ensemble was continued for 100 ns.
